# INFORMATION TO BE COLLECTED IN THE ASSESSMENT FOR PARENTS AND CHILDREN WITH “8050 PROBLEMS” IN JAPAN

**DOI:** 10.1093/geroni/igac059.2859

**Published:** 2022-12-20

**Authors:** Takako Ayabe, Yoshito Takemoto, Shinichi Okada, Sadaaki Fukui

**Affiliations:** BAIKA Women’s University, Ibaraki, Osaka, Japan; Okayama Prefectural University, Soja, Okayama, Japan; Osaka Metropolitan University, Osaka, Osaka, Japan; Indiana University, Indianapolis, Indiana, United States

## Abstract

In Japan, the “8050 problems” have become a social issue, where parents and children are parents in their 80s and children in their 50s and live together. In the case of parents and children with “8050 problems,” the parents are older, but the children are often dependent, withdrawn, and have financial problems. Recently, the number of such cases has increased in care management. The previous studies indicated that it was important to know what information was gathered in the assessment in such cases. The research was conducted from February to March of 2022 by self-administered questionnaires. The survey was mailed to all 1,410 care management centers in Osaka City. The response rate was 11.8%. The statistical analysis was an exploratory factor analysis. Six factors in the significant information in the assessment were extracted by the factor analysis: Physical, mental and living conditions of children; Parents’ physical and mental conditions; Parents’ perspectives on their lives; Parents and children’s financial situations; Living environment; and Relationships between the informal and formal supports and the parents and children. The Cronbach’s alpha for internal consistency was greater than 0.8 for each factor. In addition, the four factors were strongly associated among the six factors. In conclusions, the present study found that information about the physical and mental status, living conditions, community relations, and financial situations of the parents and children with “8050 problems” was important in the assessment of the parents and children, and that the information is closely related to each other.

